# Accurate expression quantification from nanopore direct RNA sequencing with NanoCount

**DOI:** 10.1093/nar/gkab1129

**Published:** 2021-11-25

**Authors:** Josie Gleeson, Adrien Leger, Yair D J Prawer, Tracy A Lane, Paul J Harrison, Wilfried Haerty, Michael B Clark

**Affiliations:** Centre for Stem Cell Systems, Department of Anatomy and Physiology, The University of Melbourne, Parkville, VIC, Australia; European Molecular Biology Laboratory, European Bioinformatics Institute, Wellcome Genome Campus, Hinxton, Cambridge, UK; Centre for Stem Cell Systems, Department of Anatomy and Physiology, The University of Melbourne, Parkville, VIC, Australia; Department of Psychiatry, University of Oxford, Oxford, UK; Department of Psychiatry, University of Oxford, Oxford, UK; Oxford Health NHS Foundation Trust, Oxford, UK; The Earlham Institute, Norwich, UK; School of Biological Sciences, University of East Anglia, Norwich, UK; Centre for Stem Cell Systems, Department of Anatomy and Physiology, The University of Melbourne, Parkville, VIC, Australia; Department of Psychiatry, University of Oxford, Oxford, UK

## Abstract

Accurately quantifying gene and isoform expression changes is essential to understanding cell functions, differentiation and disease. Sequencing full-length native RNAs using long-read direct RNA sequencing (DRS) has the potential to overcome many limitations of short and long-read sequencing methods that require RNA fragmentation, cDNA synthesis or PCR. However, there are a lack of tools specifically designed for DRS and its ability to identify differential expression in complex organisms is poorly characterised. We developed NanoCount for fast, accurate transcript isoform quantification in DRS and demonstrate it outperforms similar methods. Using synthetic controls and human SH-SY5Y cell differentiation into neuron-like cells, we show that DRS accurately quantifies RNA expression and identifies differential expression of genes and isoforms. Differential expression of 231 genes, 333 isoforms, plus 27 isoform switches were detected between undifferentiated and differentiated SH-SY5Y cells and samples clustered by differentiation state at the gene and isoform level. Genes upregulated in neuron-like cells were associated with neurogenesis. NanoCount quantification of thousands of novel isoforms discovered with DRS likewise enabled identification of their differential expression. Our results demonstrate enhanced DRS isoform quantification with NanoCount and establish the ability of DRS to identify biologically relevant differential expression of genes and isoforms.

## INTRODUCTION

Cellular fates and functions are underpinned by the expression of protein-coding and non-coding genes into RNA (termed the transcriptome). The expression profiles of individual genes can vary in complex ways to regulate their functional outputs. Expression of genes can be switched on or off, increased or decreased, while the RNA products (transcript isoforms) made from individual genes can also vary extensively. In humans >90% of protein-coding genes express multiple RNA isoforms via processes such as alternative transcriptional start sites, termination sites and splicing, greatly increasing the diversity of the transcriptome and proteome within cells (1,2). Expression of different genes and isoforms drive cellular differentiation programs, control cell and tissue functions and allow cells to respond to their environment (3,4). However, aberrant expression contributes to various diseases including neurological disorders, autoimmune disorders and cancer (5–7).

Understanding the dynamic transcriptome requires techniques that can identify differential expression (DE) at both the gene (DGE) and transcript isoform (DTE/DIE) levels. Differential expression analysis enables comparisons between different tissues or conditions to identify genes that play a major role in phenotype determination. Short-read RNA sequencing (RNA-seq) methodologies are well validated for identifying differential gene expression but have limitations in identifying and quantifying both known and novel alternative isoforms (8,9). This is exacerbated in complex mammalian transcriptomes which contain large numbers of highly similar transcript isoforms. A further limitation of short-read RNA-seq is the need for reverse-transcription and PCR amplification of RNA samples before sequencing, which introduces various biases (10).

Long-read sequencing techniques from Oxford Nanopore Technologies (ONT) and Pacific Biosciences have the potential to overcome many of these limitations (11,12). Long-read methods can sequence entire transcripts in a single read, potentially allowing the unambiguous identification of the expressed gene and isoform. However, initial long-read methods still required PCR and/or reverse transcription. Recently, ONT developed direct RNA sequencing (DRS), the first long-read technique to sequence native RNA molecules (13). DRS does not utilize any amplification or fragmentation steps and has the potential to quantify both genes and isoforms in an unbiased manner, while also characterising the RNA modifications and polyA tail on each RNA. Studies to date have used DRS to catalogue known and novel transcripts in yeast (14), *Caenorhabditis elegans* (15,16), *Arabidopsis* (17) and human cell lines (18,19); characterise polyA tail lengths of individual transcripts (15,18); identify allele specific gene and isoform expression (18); identify RNA base modifications (13,18,20,21) and infer RNA structure (22). Maximum read lengths for DRS were also significantly longer than for PCR-based long-read cDNA sequencing (18), demonstrating its potential to sequence long and complex RNA splice isoforms.

Identification of expression differences between samples is a standard requirement in the transcriptomics field. Therefore, DRS needs to accurately quantify RNA and identify differentially expressed genes and isoforms to become a mainstream transcriptomics technique. The unbiased nature of DRS should allow accurate quantification and has previously shown good performance on synthetic control RNAs of known abundance (13,23). However, accurate assignment of DRS reads to the correct transcript isoforms remains a challenge (19,23), which limits the accuracy of isoform quantification and downstream differential expression analyses. In addition, the ability of DRS to identify differential gene and isoform expression has largely been performed on model organisms with much simpler transcriptomes than found in mammals and/or based on expression fold changes without statistical analysis (14,16,17). Hence, there is a need for new tools that improve isoform assignment and quantification accuracy, as well as to establish the effectiveness of DRS to identify differential expression in complex organisms.

Here, we introduce NanoCount, an isoform quantification program developed for DRS, which improves isoform assignment and out-performs other quantification tools. Using a combination of synthetic spike-in control RNAs and a neuroblastoma differentiation paradigm, we demonstrate that DRS accurately quantifies genes and isoforms and is able to identify biologically relevant differential gene and transcript isoform expression. We find that variance between samples is dominated by biological differences, allowing the identification of hundreds of DE genes and isoforms despite the lower throughput of DRS. We further show that DRS can identify differential isoform usage (DIU), where switching occurs between isoforms, often in the absence of overall changes to gene expression. Lastly, we utilised NanoCount with FLAIR to discover and quantify the expression of thousands of novel isoforms in SH-SY5Y (5Y) cells, confirming the potential of DRS to help fully decipher the complex transcriptome.

## MATERIALS AND METHODS

### Cell culture

**Figure 1.**
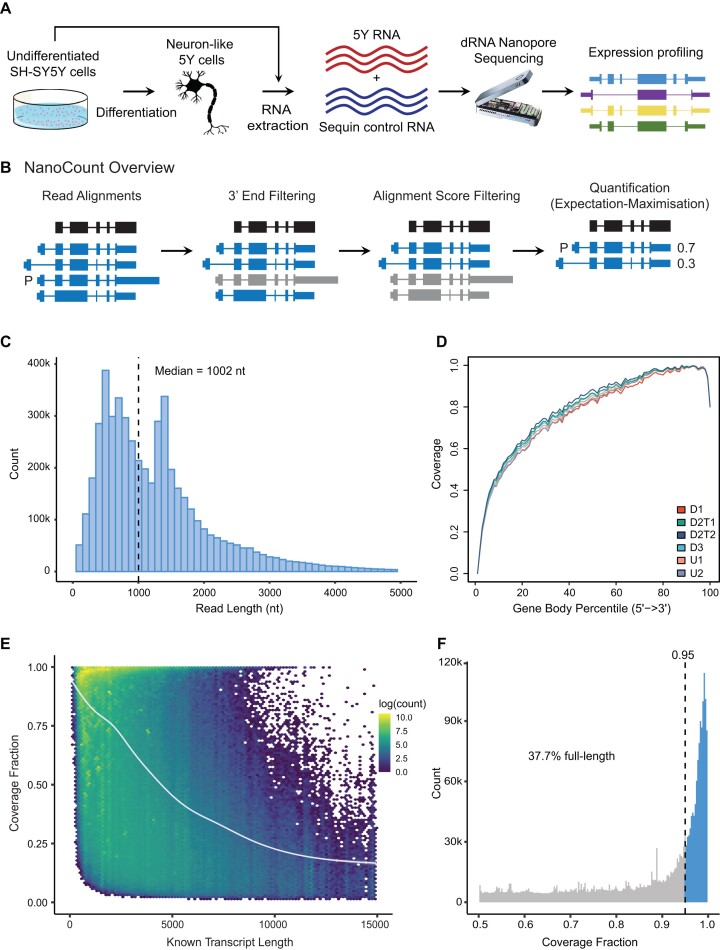
Experimental overview and DRS read metrics. (**A**) Experimental overview. Cultured SH-SY5Y cells were differentiated in triplicate and RNA extracted from undifferentiated and differentiated cells. Native polyA purified SH-SY5Y RNA was combined with ‘sequin’ spike-in RNA, prepared for DRS and sequenced on an Oxford Nanopore MinION. Reads were analysed to identify and quantify genes and transcript isoforms and their differential expression. (**B**) Overview of NanoCount steps. A nanopore read (black) and example alignments (blue) are shown. P = primary alignment. Alignments with a 3′ end >50nt from the read 3′ end are discarded (grey). Next, alignments with an alignment score (AS) <95% of the highest remaining AS are discarded. Finally, the expectation-maximisation algorithm is initiated to quantify isoforms. In this example the read count is split 0.7 to 0.3 between the two remaining alignments. The primary alignment (P) is now the remaining alignment with the highest AS. (**C**) Length of all SH-SY5Y and sequin pass reads. Dashed line shows median read length. X-axis truncated at 5 kb. (**D**) Gene body coverage of SH-SY5Y reads in each sample. Length of all genes normalised to 100 and plotted from 5′ (0) to 3′ (100). Lines show mean coverage across all genes across the length of the gene body. Lower coverage at extreme 3' corresponds to soft clipping of the first bases sequenced which often have lower phred quality. U1 & U2—undifferentiated replicates 1 & 2. D1 & D3—differentiated replicates 1 & 3. D2T1 & T2—technical replicates of differentiated replicate sample 2. (**E**) Fraction of known transcript length covered by each read (coverage fraction) compared to known transcript length. Trend line was plotted using a generalized additive model, an extension of a generalised linear model where the linear form is replaced by sum of smooth functions. (**F**) Fraction of full-length SH-SY5Y reads. Fraction of known transcript length covered by each read. Dotted line represents 95% cutoff for full-length reads. Full-length reads shaded in blue. X-axis truncated at 0.5.

Human SH-SY5Y neuroblastoma cells were cultured for use as a model of neuronal differentiation (Figure 1A) (24). Undifferentiated SH-SY5Y cells were cultured in growth media under standard conditions (5% CO_2_, 37°C) with DMEM:F12 (Sigma D6421), supplemented with 10% fetal bovine serum (FBS) (Sigma F9665), 2 mM l-glutamine and 1% non-essential amino acids. SH-SY5Y differentiations were performed in triplicate. To differentiate the SH-SY5Y cells, flasks were coated with poly-lysine. Cells were seeded at 4.4 × 10^4^ cells/cm^2^ and grown for 24 h in standard growth medium. The growth media was then exchanged for differentiation media neurobasal medium (Thermo Fisher 21103049), 2 mM l-glutamine, 1% FBS, 10% B27 supplement (Thermo Fisher 17504044) and 10 mM retinoic acid (Sigma R2625). Media was replaced after 48 h. After 72 h of exposure to 10 mM retinoic acid the media was exchanged to differentiation media without retinoic acid and cells were allowed to further differentiate for 72 h, including a media change at 48 h. RNA was then extracted from undifferentiated and differentiated cells with Tri Reagent (Thermo Fisher 15596026) and RNeasy columns (Qiagen 74106).

### Library preparation and sequencing

To prepare RNA for sequencing, Turbo DNase was added for 30 min at 37°C in a PCR thermocycler with the lid set to 50°C to remove any DNA contamination. Samples were purified with the RNeasy MinElute Cleanup Kit (Qiagen 74204) and eluted with 15 μl nuclease-free water. Samples were run on an Agilent 4200 Tapestation to ensure RNA had a RIN of >9 and quantified via Qubit (Thermo Fisher). PolyA+ purification was performed with 50 μl of NEXTflex polyA+ beads on a minimum of 40 μg of total RNA. Confirmation of rRNA removal and sample quantification were performed with Qubit and Tapestation respectively. To minimise batch effects, all samples were prepared together.

Sequencing libraries were prepared with the SQK-RNA001 kit (Oxford Nanopore Technologies, ONT) using the standard protocol, including the optional reverse transcriptase step. Two different controls were added to each sample, yeast calibration RNA (supplied by ONT) and synthetic sequin V2 spike-in RNAs (25). The calibration RNA is from the yeast enolase II (YHR174W) gene and is 1.3 kb in length. RNA sequins provide a quantitative and qualitative reference to enable transcriptome analysis. Sequins are synthetic spliced RNA transcripts for the investigation of gene and isoform quantification, alternative splicing and differential expression. RNA sequins consist of 160 isoforms from 76 artificial genes that vary in concentration over 4 orders of magnitude. Sequin controls are available in two mixes, Mix A and Mix B, which contain the same synthetic RNA isoforms but at different concentrations. Sequins were added to each sample at a concentration of 6%. Undifferentiated samples (U1, U2) contained sequin Mix A, and differentiated samples (D1, D2TR1, D2TR2, D3) contain Mix B. Sample input was 352.5 ng of polyA+ RNA and 22.5 ng of sequin RNA. Libraries were sequenced on the ONT MinION using R9.4.1 flow cells and MinKNOW (v18.01.6) to generate FAST5 files. FAST5 files were basecalled with Guppy (v3.4.5) (ONT) to create summary text files and FASTQ files of the reads.

### Sequencing metrics and quality control

Initial data analysis was performed to gather general metrics on sequencing performance. All analyses were performed on pass reads (quality (*Q*) score ≥ 7). To ensure the sequencing was of high quality, EPI2ME (ONT) was used to determine the sequencing accuracy of the yeast calibration RNA. All subsequent analyses were performed on pass SH-SY5Y and sequin reads (hereafter referred to as reads). Overall data characteristics including median read length; median quality score; longest read length; total number of reads; read length and quality scores over run time were performing using NanoPack (26) and pycoQC (27).

### Genome and transcriptome alignment

FASTQ files containing ONT pass reads were aligned to the human (GRCh38) (28) and synthetic (25) sequin genome and transcriptome using minimap2 (v2.17) (29). The genome alignments were performed using the splice-aware mode of minimap2 *-ax splice -uf -k14* as recommended. Alignments to the GENCODE (v31) and sequin transcriptomes were performed using the long-read mode for ONT data *-ax map-ont -N 100* to retain multiple secondary alignments. When two alignments both have the same scores, minimap2 assigns the primary one at random. To maximise the likelihood of identifying the correct isoform of origin, we retained many secondary alignments that were later filtered during quantification.

### Development and benchmarking of NanoCount

We developed NanoCount for transcript quantification of nanopore direct RNA reads (Figure 1B). NanoCount has been designed to be compatible with minimap2, but is potentially compatible with alternative aligners that can output: (i) multiple alignments per read; (ii) a metric equivalent to the minimap2 alignment score (AS). NanoCount requires a sorted BAM file of reads aligned to the transcriptome as input. The first steps in NanoCount filter reads based on optimised (customisable) criteria and defines the best alignment per read (detailed below). After these optional filtering steps, NanoCount uses an expectation-maximisation (EM) algorithm to estimate transcript abundances. Firstly, an initial read/transcript scores hash table is generated (compatibility index). For each alignment we define an initial score corresponding to the fraction of the number of alignments per read. This means that alignments from multimapping reads are down weighted. Secondly, the EM loop is initiated, which starts by calculating the transcript abundance. For each transcript the abundance is defined as the sum of scores extracted from the current compatibility index for alignments mapping to that reference, divided by the total score for all the alignments. The compatibility index is then updated as follows: for each alignment we divide the current compatibility score by the sum of abundance scores of transcripts for which the corresponding read aligns to. This increases the scores of alignments mapping on abundant transcripts which are more likely to be true positives. These two steps are repeated until the convergence is reached. The convergence is defined as a low cumulative transcript abundance difference between two successive EM rounds (customisable). Finally, NanoCount computes estimated counts and normalised TPM values based on the final transcript abundance table.

NanoCount incorporates filtering steps to improve transcript quantification. By default these are set to provide highly accurate results based on our benchmarking (Supplementary Table S1) but are user customisable. NanoCount initially performs basic DRS filtering steps that discard: reads with a low aligned fraction (<0.5, customisable), short alignments (<50 nt, customisable) and negative strand alignments. Due to the mechanism of DRS, sequencing begins at the 3′ end (polyA tail) of RNAs, therefore the alignment 3′ end should be close to the transcript 3′ end. NanoCount includes a 3′ filtering threshold (MAX_DIST_3_PRIME; default: 50 nt) for the maximum nucleotide distance of the alignment end to the 3′ transcript end. NanoCount also includes an alignment score (AS) threshold (SEC_SCORING_THRESHOLD; default: 0.95) which a secondary alignment must meet to be considered valid. The default requires a secondary alignment score to be at least 95% of the highest alignment score for that read. Full documentation is available at: https://a-slide.github.io/NanoCount/. NanoCount optionally outputs a BAM file of the alignments remaining after filtering. We used NanoCount (v1.0.0) with the above default parameters to generate all isoform quantification results with the following command: *NanoCount -i file.bam -o output.tsv -b output.bam –extra_tx_info*. The program is available to download from: https://github.com/a-slide/NanoCount/.

### Full-length transcript identification

A custom script was used to extract data from transcriptome BAM files and identify full-length transcript isoforms (https://github.com/josiegleeson/BamSlam). Coverage fractions were calculated by dividing the observed length (alignment length) by the original known transcript length for each read's best alignment (highest AS) defined by NanoCount. Reads were required to cover at least 95% of their annotated transcript to be classed as full-length. To assess the relationship between secondary alignments per read with coverage fractions, we used each read's best alignment and corresponding coverage fraction. This value was then compared to that read's total number of secondary alignments to produce correlations.

### Comparison of isoform quantification methods

The performance of NanoCount (v1.0.0) was benchmarked against four other transcript quantification programs that are compatible with DRS: Salmon (v0.13.1), StringTie2 (v2.1.5), FLAIR (v1.4.0) and LIQA (v1.1.16) (30–33). We used the Spike-In RNA Variant Control (SIRV) mixes E0 and E2 (SRR6058583) for our benchmarking dataset (13). Each SIRV mix contains 69 transcripts from seven human-like genes. Mix E0 contains all transcripts at equal concentrations, whereas mix E2 contains the transcripts in varied concentrations that extend over two orders of magnitude. There are three annotations of SIRV transcripts; a complete annotation of all transcripts (C), an insufficient annotation which is missing transcripts that are present in the mix (I) and an over annotation which contains additional transcripts that are not present in the mix. For mix E0, the quantification program that reported the lowest coefficient of variance (standard deviation/mean) was considered the best as all transcripts should be given the same count. For mix E2, performance was measured by Spearman's rho correlation between known and measured abundance. Performance data on each program is contained in Supplementary Table S1. Salmon was run in three separate modes: default; disabling the error model (–noErrorModel); and disabling both the error model and length correction (–noErrorModel, –noLengthCorrection). The correlations were highest when disabling the error model, so this mode of Salmon was included for benchmarking. StringTie2, FLAIR and LIQA were run in their default modes.

### Gene and isoform quantification

FeatureCounts was applied (v1.6.5) (34) to the human or sequin genome alignments along with GENCODE (v31) annotations to calculate gene counts with the parameters *-L –primary*. NanoCount was used for isoform quantification on BAM files of all alignments as described above. Sequin genes and isoforms are present at known concentrations over a 32 773 concentration fold range (genes), and a 229 409 fold range (Mix B isoforms) and with known fold changes between Mix A and Mix B. To assess the DRS quantitation, Mix B sequin counts at the gene and isoform level were compared to observed counts. Only detected sequins (count>0) were included in this analysis. Segmental linear regression (SLR) was used to identify the sequin concentration where quantitative measurement began. Criteria for breakpoint were (i) multiple sequin genes/isoforms at concentration; (ii) ≥50% detection across all replicates at concentration; (iii) 95% confidence interval of slope before breakpoint should include 0; (iv) optimal or near optimal SLR goodness of fit. All sequin Mix B genes and isoforms detected in at least one replicate were used to calculate Spearman correlations. To assess accuracy for detecting fold changes, known fold changes between Mix A and Mix B were compared to observed fold changes. Slopes were determined by linear regression.

### Differential expression analysis

Differential expression analysis was performed with DESeq2 (v1.24) (35). Normalised counts for the two technical replicates, Diff2TR1 and Diff2TR2, were averaged prior to analysis to produce Diff2 so as not to falsely increase the statistical power between groups. The counts from featureCounts and NanoCount were input for gene and isoform level analysis respectively. Count matrices were filtered to remove very lowly expressed features (≤5 in total for each gene/isoform). Counts were normalised for sequencing depth within DESeq2 prior to statistical analysis. Log_2_ fold changes and adjusted *P*-values (using the Benjamini–Hochberg method to correct for multiple testing) were calculated for each annotated gene or isoform and used to determine statistical significance. A regularised log transformation was subsequently performed on the normalised counts for visualisation. The PCA and volcano plots were made using the following code: https://gist.github.com/stephenturner/f60c1934405c127f09a6.

Differential isoform usage (DIU) analysis was performed using an R package IsoformSwitchAnalyzeR (36). The isoform counts from NanoCount were input, along with the annotation and transcriptome files. The DIU statistical analysis was performed within IsoformSwitchAnalyzeR with DEXSeq (v1.32) (37,38) to identify exons (used to infer transcript isoforms) present in different proportions between groups. Counts were filtered to remove single isoform genes and a gene and isoform expression cutoff of 5 was included to filter out lowly expressed features. A cutoff fraction of 0.1 was used as the minimum difference in isoform fraction between conditions to further increase stringency. DEXSeq normalises counts and outputs a table of isoform fractions and their adjusted *P*-values for switches. The coding potential and protein domains of transcripts were then predicted as part of IsoformSwitchAnalyzeR using CPAT and PFAM respectively (39,40). Premature termination codons and nonsense-mediated decay sensitivity were also predicted as part of the workflow, which enabled the functional consequences of isoform switches between conditions to be predicted and plots to be produced of isoform switches. Known isoform fractions in sequin data were calculated by dividing each isoform concentration by its total gene concentration within each condition. To compare isoform switches with DGE and DTE in the same genes we used consistent counts data to calculate adjusted *P*-values. DTE results from DESeq2 (described above) were input directly, while DGE was performed with DESeq2 after collapsing isoform counts to gene counts with IsoformSwitchAnalyzeR.

### Novel transcript identification

FLAIR (v1.4) was used on the human and sequin primary genome alignments (BAM converted to BED12 files using FLAIR script) for novel transcript identification (32). Samples were first corrected using genome annotations and with the *-n* flag enabled to keep read strands consistent after correction. The two separate corrected PSL files for human and sequin data were then collapsed into high-confidence isoforms. This required that isoforms were represented by at least five full-length reads (80% coverage and covering 25 bp of the first and last exon). The FLAIR ‘–trust_ends’ option was enabled for long reads and a BED file of CAGE peak data (41) was input to only retain isoforms with 5′ ends within 100nt of known transcriptional start sites.

The collapsed isoform GTF files were compared to annotated transcripts using gffcompare (v0.11.2) (42). Gffcompare assigns each transcript a class code based on how it compares to annotated reference transcripts. The class codes were grouped into the following categories. Known: full ( = ) or partial (c) intron chain match to annotated transcript isoform. Novel isoforms of known genes: novel splice isoform (j), splice chain match with additional terminal exon(s) (k), retained intron isoforms (m,n). Novel transcripts potentially representing novel genes or transcriptional units: intronic transcript (i), antisense transcript (x), intergenic transcript (u), 5′ distal overlapping RNA (y), other exonic overlap (o). Other: including RNA fragments and potential sequencing artifacts (p,e,s,r). In order to create an updated transcriptome and annotation which included high-confidence novel isoforms, the FLAIR outputs were filtered to only include novel multi-exon isoforms (retaining isoforms from class codes: j, k, m, n, I, x, u, y, o). This subset FASTA/GTF file from FLAIR was then combined with the original GENCODE (v31) FASTA/GTF to create the updated transcriptome and annotation. The 5Y reads from each sample were then re-mapped to this transcriptome with minimap2 and quantified with NanoCount as previously described. This new count file was then used as input for differential isoform expression and usage analyses as previously described.

### Novel isoform validation

First-strand cDNA was synthesized from 2 ug of total SH-SY5Y RNA using the Maxima H Minus Reverse Transcriptase protocol (Thermo Fisher EP0751) in accordance with manufacturer's instructions. Primer pairs were designed using primer3 (https://bioinfo.ut.ee/primer3/) and are listed in Supplementary Table S2. PCR conditions were 30 cycles of: 95°C denaturation for 30 s, 60°C annealing for 30 s and 68°C extension for 30 s. PCR products were run on a 1.2% agarose gel to validate amplification. PCR products were prepared for sanger sequencing using ×1.8 ampure bead cleanup and sequenced using Applied Biosystems 3730XL DNA Analyser. Additionally, we performed ONT long-read cDNA sequencing on two PCR amplicons. Sequencing libraries were prepared with the SQK-LSK109 kit and samples were barcoded using EXP-PBC096 (ONT). Libraries were sequenced on the ONT GridIon using FLO106 flow cells and MinKNOW (v20.10.6). Pass reads (quality (*Q*) score ≥ 7) were mapped to the genome using minimap2 (v2.17) and alignments were assembled into transcripts for visualisation on the UCSC browser

## RESULTS

To examine the ability of DRS to identify differential expression from a complex mammalian transcriptome, we utilised the well characterised differentiation of the human neuroblastoma SH-SY5Y (5Y) cell line into neuron-like cells. Native polyA+ RNA from duplicate samples of undifferentiated and triplicate samples of differentiated 5Y cells were sequenced on an Oxford Nanopore MinION (Figure 1A). In addition, a technical replicate of one differentiated sample was prepared to examine variability due to library preparation and sequencing. Synthetic RNA ‘sequin’ spike-in controls (25) (see methods) were included in all samples to provide positive controls for gene and isoform identification, quantification and differential expression. Sequin RNAs vary in abundance over >4 orders of magnitude and come in two mixes, each mix contains the same synthetic RNA isoforms but their concentrations are offset by known amounts. Mix A was added to undifferentiated 5Y RNA, while Mix B was added to RNA from differentiated samples. A yeast calibration RNA (see methods) was also included to enable initial quality control of sequencing.

### Sequencing metrics and read assignment

DRS generated 6.5 million reads in total, of which 4.4 million (∼68%) were pass reads with a quality score >7. Yeast calibration RNA generated 325k pass reads which had a median read length of 1.3 kb, which was consistent with the known length of this control and a median accuracy of 91%. There were ∼4.1 million pass reads from 5Y cells and RNA sequins (hereafter referred to as “reads”). Reads had a median length of 1004 nucleotides and a median quality score of 10 across all samples (Figure 1C, Table 1).

**Table 1.** Direct RNA sequencing alignment metrics (pass reads)

	Undiff1	Undiff2	Diff1	Diff2TR1	Diff2TR2	Diff3	Overall
Number of reads	468 790	778 037	518 176	806 067	867 371	634 226	4 072 667
Median read length (nt)	989	1009	922	994	1041	1042	1004
Median read quality score	9.8	10.2	9.9	10.0	10.0	9.9	10.0
Longest alignment length (nt)	13 439	15 081	12 600	13 739	13 624	14 362	15 081
Reads aligned to genome (%)	97.5	97.8	97.8	98.1	98.2	98.1	98.0
Reads aligned to transcriptome (%)	97.7	97.8	98.0	98.3	98.4	98.3	98.2

Reads from each sample were aligned using minimap2 (29) to the human and sequin reference genome and transcriptome. The aligned lengths had an overall median of 991 nucleotides (Supplementary Figure S1A), demonstrating almost the entire length of the reads aligned. Ninety eight percent of reads aligned to the genome and transcriptome, meaning almost all reads were assigned to a gene and transcript isoform (Table 1). Most reads only had a single primary genomic alignment. Similar to previous findings (19), >70% of reads had one or more secondary transcriptomic alignments, largely reflecting the increased difficulty of assigning reads to specific isoforms (Supplementary Figure S1B).

To improve isoform assignment and subsequent isoform quantification we developed NanoCount (see Methods). NanoCount performs DRS specific filtering steps to improve transcript assignment prior to quantifying DRS reads using an expectation-maximisation algorithm (Figure 1B). NanoCount filters alignments based on two key parameters, read 3′ end position and alignment score (AS). As DRS reads always start at the RNA 3′ polyA tail, there should be close agreement between the 3′ end of a read and the 3′ end of the transcript it aligns to. In addition, alignment scores identify the best matching alignments and allow separation of close matching putative primary and secondary alignments from lower quality secondary alignments. After testing a number of parameters and parameter combinations (Supplementary Table S1) we used NanoCount to discard read alignments with 3′ ends >50nt away from the transcript 3′ end and secondary alignments with an AS <95% that of the best alignment (highest AS), as these likely represent misalignments or reads from unannotated isoforms. Examples of how NanoCount filters alignments are shown in Supplementary Figure S2.

Filtered read alignments from NanoCount greatly improved transcript assignment, increasing reads with a single primary alignment two-fold to 56% and decreasing reads with five or more secondary alignments by >80% (Supplementary Figure S1B). We identified expression of 17 263 human genes (65% of protein coding genes) and 41 703 human isoforms (41% of protein coding isoforms), as well as 42 (55%) and 70 (44%) sequin genes and isoforms.

### Genome and transcriptome coverage

One of the main advantages of nanopore sequencing is the potential to generate long reads that comprise full-length transcripts. However, both RNA degradation and ONT software limitations reduce the extent to which DRS reads represent full-length transcripts (18). Sequin RNAs are in-vitro transcribed and so are not vulnerable to degradation by cellular RNases during RNA extraction. As such they should provide some indication of how much degradation is present in cellular RNA (even when RNA integrity appears high) versus how many incomplete transcripts are due to degradation during library preparation and/or sequencing limitations.

We examined read coverage across gene bodies. DRS requires an intact 3′ end, which is sequenced first (13) and we observed corresponding high coverage at the 3′ end (Figure 1D). Gene body coverage then progressively decreased towards the 5′ end, consistent with previous studies (19,43).

To assess the fraction of transcripts covered by reads and the proportion that represent full-length transcripts a coverage fraction was calculated. We utilised the best alignment for each read (to GENCODE (v31) or the sequin transcriptome) after NanoCount filtering. We defined coverage fraction as the observed transcript length (alignment length) divided by the original known transcript length. The median coverage fraction for 5Y RNA was 0.88 (0.97 for sequin RNA), demonstrating that most reads covered a high proportion of the original RNA transcript. Alignments to longer transcripts were less likely to be full-length, consistent with previous studies (14) (Figure 1E, Supplementary Figure S3A). In addition, the number of secondary alignments correlated with the coverage fraction (–0.23 for 5Y and –0.70 for sequins) (Supplementary Figure S3B,C), confirming that improved sequencing coverage at the 5′ end would further decrease secondary alignments and improve quantification.

We classified alignments that covered at least 95% of their assigned transcript isoform as full-length (14). In total, 53% of sequin reads were full-length compared to 38% of 5Y reads (Figure 1F). In comparison, using the primary alignments from minimap2 without NanoCount filtering gave a median 5Y coverage of 0.74, with 46% of sequin reads and 29% of 5Y reads being full-length. These results demonstrate the benefit of read filtering and improved transcript assignment. However, while a significant proportion of reads are full-length, there is considerable room for improvement through sequencing software advances and by preventing degradation during RNA extraction and library preparation.

### Accurate expression quantification of DRS with NanoCount

**Figure 2.**
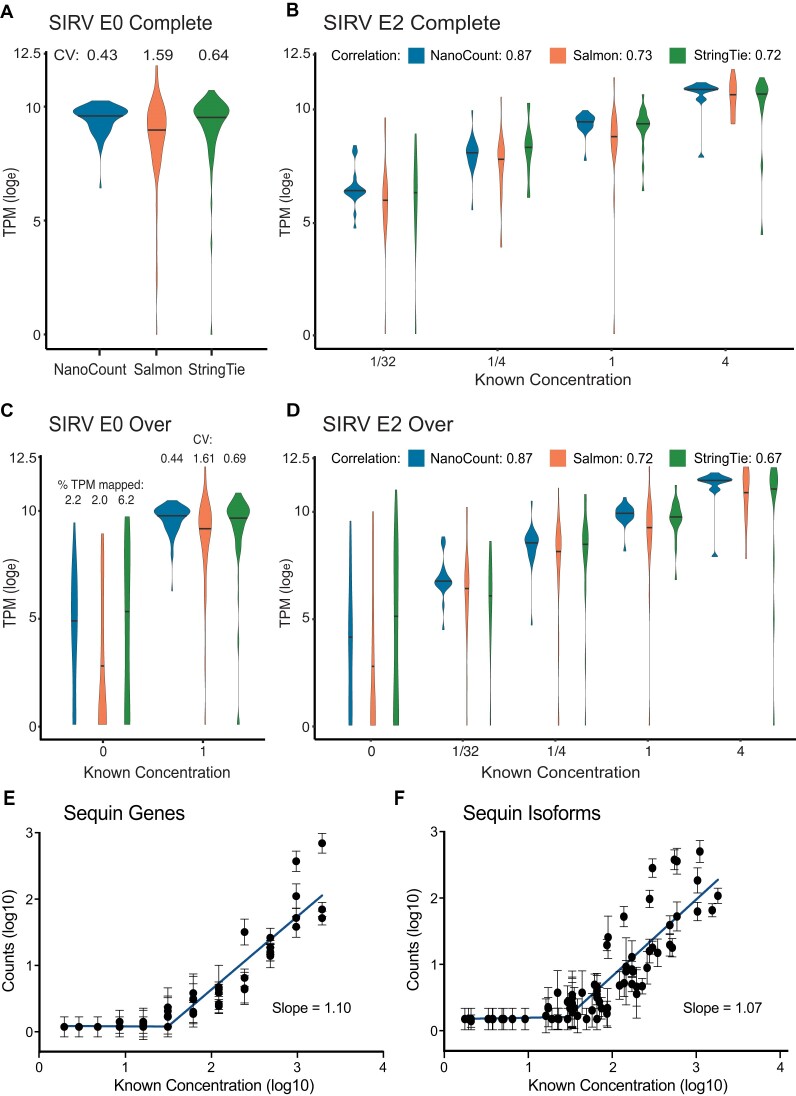
Comparison of methods for quantifying DRS spike-in controls. (A–D) Comparison of NanoCount, Salmon and StringTie2 for quantification of SIRV spike-in isoform mixes. Median lines plotted. SIRV isoform expression quantified as TPM(log_e_), i.e.: log_e_(TPM + 1). (**A**) SIRV Mix E0 Complete annotation (C) isoforms (coefficient of variation (CV) of TPMs shown). (**B**) SIRV Mix E2 Complete annotation (C) isoforms (Spearman's r correlation shown). (**C**) SIRV Mix E0 Over annotation (O) isoforms. TPM coefficient of variation shown for isoforms with a known concentration of 1 fmol. For false positive isoforms with a known count of 0 TPM distribution is plotted and the % of TPMs mapped to 0 count isoforms shown. (**D**) SIRV Mix E2 Over annotation (O) isoforms (Spearman's r correlation shown). (E, F) Quantification of sequin genes (**E**) and transcript isoforms (**F**). Comparison of known sequin Mix B abundance (original concentration in attomoles/ul) to measured counts. Counts and concentrations transformed log_10_(*X* + 1). Mean and standard deviation plotted. *N* = 4. Trend line = segmental linear regression with breakpoint at 1.49 (genes) and 1.44 (transcripts) performed on log_10_ transformed data.

We tested the performance of NanoCount against other transcript quantification programs, including Salmon, StringTie2, FLAIR and LIQA (19,23,32–33,33), using SIRV RNA spike-in DRS data (Materials and Methods). In the SIRV mix E0 where all isoforms are present in the same concentration, NanoCount quantification consistently displayed the least variability between measured isoform abundances (Figure 2A, C, Supplementary Figure S4, Supplementary Table S1). This was maintained for the over annotated (O) and insufficiently annotated (I) sets, which represent more realistic situations where either only a subset of annotated transcripts are expressed (O), or, un-annotated novel transcripts are present in the data (I). For SIRV mix E2 where transcript concentrations vary, NanoCount also had the best overall performance (Figure 2B, D, Supplementary Figure S4, Supplementary Table S1). The improved NanoCount performance, especially in the over (O) and insufficient (I) annotations, suggests alignment filtering is an important step for accurate transcript assignment and quantification for genes with high levels of isoform complexity. Like Salmon and StringTie, NanoCount has a very short run-time, with all three programs completing isoform quantification within 10 s (real-time) (Supplementary Table S1).

To confirm the quantitative accuracy of DRS and its potential to detect differential expression we used sequin spike-ins, which are better suited for testing DE. Quantification was performed with featureCounts for alignments to the genome (34), and NanoCount for alignments to the transcriptome respectively, and compared with known sequin input concentrations (Figure 2C, D, Supplementary Table S3). Measured sequin abundance was highly correlated with known abundance at the gene and isoform level (Spearman's correlation of 0.96 and 0.90 respectively, both *P* < 0.0001, two-tailed). Results showed DRS could detect but not quantitate sequins at very low concentrations due to sequencing depth. Therefore a segmental linear regression was utilised to help identify the concentration where quantitative measurement began. This revealed a slope close to 1 above the cutoff for genes and isoforms. These findings demonstrate the ability of DRS to accurately quantify detected genes and isoforms and the improved isoform quantification produced by NanoCount. Therefore, we utilised NanoCount quantification for all subsequent transcript-level analysis.

### Identification of differential expression with direct RNA sequencing

**Figure 3.**
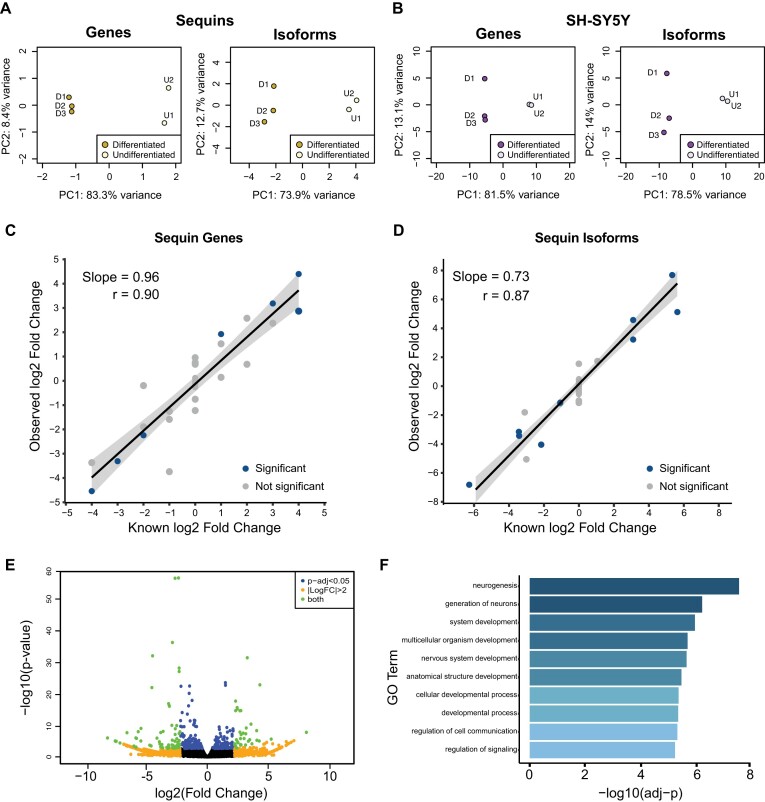
Identification of differential gene and isoform expression. (A, B) Principal component analysis (PCA) of sequin (**A**) and SH-SY5Y (**B**) gene and isoform expression between undifferentiated and differentiated SH-SY5Y cells. All plots show the first two principal components. SH-SY5Y shows endogenous expression only. Sequins were added to undifferentiated (Mix A) and differentiated (Mix B) SH-SY5Y RNA and plots reflect measured abundance differences between the sequin mixes. (C, D) Quantification of fold changes between Mix A and Mix B sequin genes (**C**) and isoforms (**D**). Comparison of known fold changes in abundance with measured fold changes from sequencing. Sequins with significant differential expression in blue, not significant in grey. Trend line shows slope from linear regression. Shaded grey area shows 95% confidence interval for regression slope. Correlation (r) is spearman correlation. (**E**) Volcano plot of differential isoform expression between undifferentiated and differentiated SH-SY5Y cells. An adjusted *P*-value <0.05 (from DESeq2) was considered significant for differential expression. FC = fold change. (**F**) Gene ontology (GO) terms most associated with differentially expressed genes upregulated during SH-SY5Y differentiation. *P*-values adjusted using Bonferroni correction for multiple testing.

We next asked if DRS could detect systematic expression level changes between conditions using principal component analyses (PCA). Comparison of Mix A and B sequins demonstrated sequin mixes separated on PC1 (Figure 3A, Supplementary Figure S5), which explained a very high proportion of the variance (83% at the gene level and 74% at the transcript isoform level). Similarly, endogenous 5Y samples separated by differentiation state along PC1, which explained 82% of the variance at the gene and 79% at the isoform level (Figure 3B). Technical replication produced almost identical samples, further confirming little technical variability from library preparation and sequencing (Supplementary Figure S5 and S6). Together the sequin and 5Y results illustrate that DRS can robustly identify differences in expression between samples at both the gene and isoform level and that the measured expression changes are reflective of the biological changes between samples.

We performed DGE and DTE using DESeq2 (35). We identified seven sequin genes and nine sequin isoforms as differentially expressed (adj-*P* < 0.05). All were true positives suggesting high accuracy in the results (Figure 3C, D, Supplementary Table S4). There are 47 sequin genes and 83 sequin transcripts potentially detectable as differentially expressed (fold change ≠ 0), however only 18/24 of these genes and transcripts passed our filtering thresholds for analysis. This suggests the high specificity, but lower sensitivity may be due to low overall read counts. To examine if DRS accurately identified changes in sequin concentrations between Mix A and B, even if significance was not reached, we examined the correlation between observed and known log_2_ fold changes of sequin genes and isoforms and also performed a linear regression to estimate the quantification accuracy of fold change measurement (Figure 3C, D). Correlations between observed and known log_2_ fold changes were high, 0.90 for genes and 0.87 for isoforms, and the slopes were 0.96 for genes and 0.73 for isoforms (both significant at *P* < 1 × 10^–12^). The significant linear relationships and strong correlations at both the sequin gene and isoform level demonstrates DRS accurately detected changes in expression between groups. The lower values for isoforms may be due to lower reads counts for isoforms than genes and the increased difficulty in unambiguously assigning reads to specific isoforms (Supplementary Figure S1B).

Applying the DE analysis on 5Y samples identified 231 annotated genes and 333 transcript isoforms as DE between undifferentiated and differentiated samples (adj-*P* < 0.05) (Figure 3E, Supplementary Figure S7, Supplementary Table S5). Genes significantly upregulated in differentiated samples (*n* = 118) were used in a gene ontology analysis using PANTHER (44). The most significant GO terms were predominantly associated with neuronal development and developmental processes more generally, with the GO term ‘neurogenesis’ (GO:0022008) the most associated (adj-*P* = 2.9 × 10^–8^) with differentiated 5Y cells (Figure 3F). GO analysis for upregulated isoforms (*n* = 197) gave similar results, including terms associated with nervous system development (Supplementary Table S6). These results validate the ability of DRS to identify biologically relevant changes in gene expression.

### Differential isoform usage (DIU) analysis between samples

Along with DE of genes and transcript isoforms, changes in isoform usage (DIU) can also be physiologically relevant and reveal important distinctions between cell types and in disease (45). DIU involves isoform switches or changes in isoform proportions and can occur even when there is no change in expression at the gene level. As the results of DTE often largely reflect those of DGE, complexity at the isoform level can be masked (46). Our results demonstrated DRS can perform quantitative sequencing of full-length isoforms and hence should be well suited to revealing this complexity.

**Figure 4.**
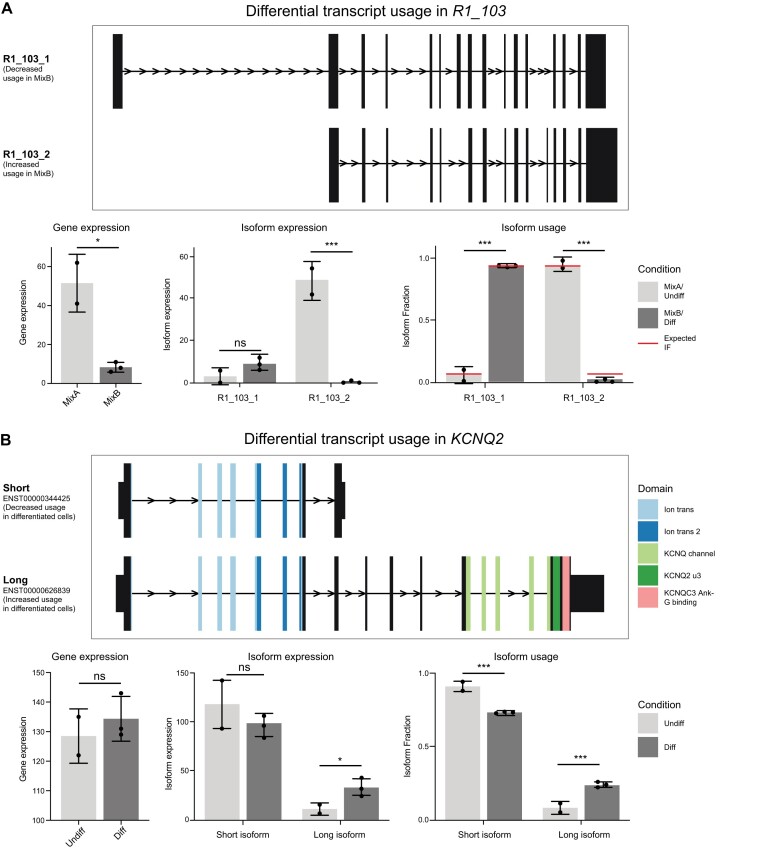
Differential isoform usage of control and endogenous genes. Identification of DIU in the synthetic sequin gene *R1_103* (**A**) and the potassium channel *KCNQ2* (**B**). Top: structure of expressed isoforms. Arrows show direction of transcription. Narrow lines show intronic regions (not to scale). Exons displayed as boxes. Taller exonic boxes are coding regions, shorter boxes are 5′ and 3′ UTR regions. Colours represent identified protein domains. Below: graphs display gene and isoform expression and the fraction of expression corresponding to each isoform in Mix A/Undifferentiated and Mix B/differentiated samples. Red lines show known (expected) sequin isoform fractions in Mix A and B. ns = not significant, * < 0.05, ** < 0.01, *** < 0.001.

Transcript counts were used to identify isoform switching using DEXSeq within the IsoformSwitchAnalyzeR package (36). After filtering out lowly expressed genes and isoforms, and isoform fraction changes of less than 0.1, 5513 5Y isoforms and 28 sequin isoforms were included for analysis. We identified 27 endogenous genes (44 isoforms) and one sequin gene (adj-*P* < 0.05) that contained isoform switches between undifferentiated and differentiated 5Y cells (Supplementary Table S7). The sequin isoform (adj-*P* = 6.5 × 10^–18^) was a true positive for DIU, with a similar observed difference in isoform fractions (0.90) to the known difference (0.88) (Figure 4A). Only three genes and 19 isoforms with DIU also showed DGE or DTE respectively, confirming how complexity at the isoform level can be masked and the importance of separately identifying DIU. To identify the potential consequences of isoform switching, the open reading frames, coding potential and protein domains were predicted for each isoform. Consequences ranged from changes to non-coding 5′ transcriptional start sites and 3′UTRs to alterations in coding regions and protein domains or switches between coding and non-coding isoforms.

DIU identified in endogenous 5Y genes included examples which matched the known biology and expression changes in neuroblastoma cells. *KCNQ2* forms a multimeric potassium channel and expresses multiple mRNA isoforms encoding functionally variant proteins. The short isoform ENST00000344425 (protein isoform 6) lacks much of the cytoplasmic C-terminal region and can alter channel properties to suppress the potassium current (47). It is the dominant isoform in undifferentiated IMR-32 neuroblastoma cells and also expressed in developing brain. In contrast, the long isoform ENST00000626839 (protein isoform 2), is the major protein isoform in adult brain and up-regulated in differentiated IMR-32 cells (47). While we find no overall change in the expression level of *KCNQ2* in 5Y cells, we identified an expression shift from the short to the long isoform upon differentiation, as would be expected from previous findings (Figure 4B). Together these results demonstrate long-read DRS can identify and quantify biologically relevant changes in isoform usage.

### Discovery and differential expression of novel isoforms

**Figure 5.**
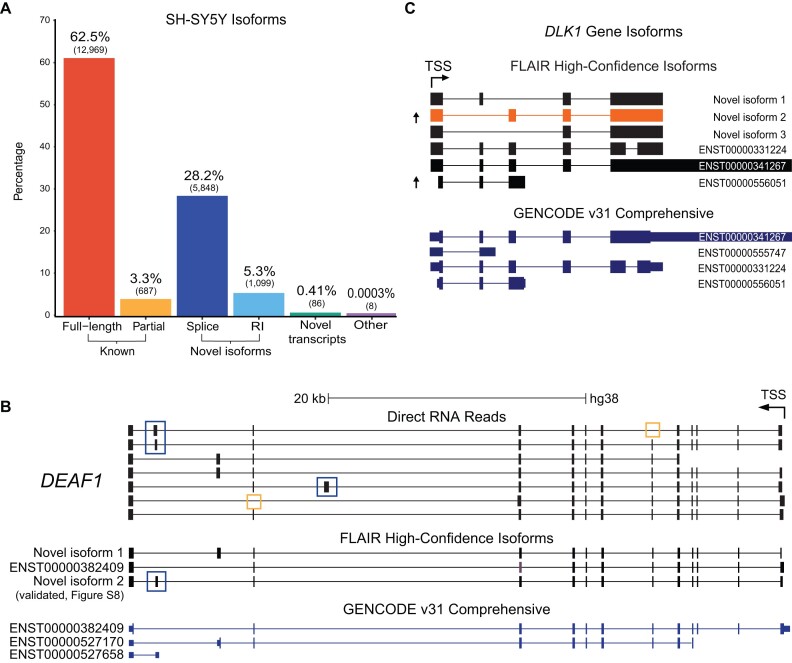
Identification and quantification of novel isoforms with FLAIR and NanoCount. (**A**) FLAIR annotation of SH-SY5Y isoforms compared with known annotations using gffcompare (42). The gffcompare class codes were grouped into the following four categories: known, transcripts that are full-length or partial-length exact matches to existing annotations; novel isoform, transcripts from known genes with novel exon junctions or retained introns (RI); novel transcript, novel intergenic, antisense or intronic transcripts potentially representing novel genes; other, possible artifacts and RNA fragments. Number of isoforms in each category shown in brackets. (**B**) UCSC Genome Browser screenshots of *DEAF1* isoforms identified by FLAIR compared to known GENCODE annotations and selected raw nanopore DRS reads. The novel *DEAF1* FLAIR isoform validated with a combination of Sanger and ONT PCR-cDNA sequencing is labelled, see Supplementary Figure S8 for validations. Novel exons and exon skipping shown in blue and yellow boxes respectively. (**C**) UCSC Genome Browser screenshots of *DLK1* isoforms identified with FLAIR compared to known GENCODE annotations. Arrows indicate significant differential expression of isoforms upregulated in differentiated samples after quantification with NanoCount. Novel isoform 2 (orange) is the most significant differentially expressed novel isoform and shows a novel 3′ terminal exon and exon skipping. TSS: transcription start site.

A key advantage of long-read sequencing is its ability to discover novel transcript isoforms (15,18). We used FLAIR (32) with stringent settings (see Methods) to identify a high-confidence set of 20 740 unique human isoforms (Figure 5A, Supplementary Table S8). Comparison to reference annotations using gffcompare (42) demonstrated the most abundant category (62.5%) was comprised of full-length matches to annotated isoforms (Supplementary Table S9). Novel isoforms of known genes (33.5%) were also frequent and largely consisted of novel splice isoforms as opposed to transcripts with retained introns. Previous long-read studies using DRS with FLAIR observed higher percentages of novel isoforms (∼50%) but used less stringent settings (18,19).

We validated an example novel isoform identified by FLAIR in *DEAF1*, a transcription factor essential for nervous system development (48,49) (Figure 5B). In agreement with the individual DRS reads, FLAIR identified the canonical isoform (ENST00000382409) and also provided evidence that two other GENCODE transcripts are incomplete isoforms that instead use the standard 5′ initiation site. We validated the most abundant novel *DEAF1* isoform and confirmed inclusion of an exon similar to that from ENST00000527658 (Supplementary Figure S8).

Given the high error rate of DRS, we used the sequin data to estimate what proportion of novel isoforms identified with FLAIR might be false positives, as all sequin isoforms are annotated and therefore should be classed as known. FLAIR identified 48 unique sequin isoforms, only one of which was classed as novel, suggesting stringent FLAIR settings produce accurate isoforms from direct RNA data (Supplementary Table S9).

To quantitate and identify DE of novel 5Y isoforms we incorporated our high-confidence FLAIR novel isoforms into an updated transcriptome annotation, re-mapped the reads and quantified expression using NanoCount, identifying the expression of 5421 novel isoforms. We then applied the same DTE analysis to detect DE of known and novel transcripts isoforms. We found 326 differentially expressed isoforms, 40 of which were novel (Supplementary Table S10). The most significant novel transcript showing DE was an isoform of the *DLK1* gene, which was upregulated in differentiated cells (Figure 5C). Measured fold changes for the 333 isoforms DE in the original analysis were highly correlated (*R*^2^ = 0.99) with the updated analysis and 247 isoforms were DE in both, demonstrating high consistency between the analyses and supporting the inclusion of novel isoforms via this method.

Finally, we also investigated switching (DIU) of novel isoforms using the process described previously. We found one gene that contained a significant novel isoform switch. A novel isoform of the *RET* proto-oncogene showed a decreased fraction of expression (–20%) in differentiated 5Y cells, whereas a known isoform of this gene had an upregulated fraction of expression (+12%) (Supplementary Table S7). These results demonstrate that the combination of FLAIR and NanoCount enables the identification of differential expression in novel isoforms.

## DISCUSSION

To enable wide uptake in the transcriptomics field, DRS needs to quantify RNA expression accurately and identify differential expression between tissues, developmental stages and disease states. To facilitate this process, we developed NanoCount for the accurate quantification of transcript isoforms and performed an in-depth analysis of DRS using synthetic spike-in RNAs and human SH-SY5Y (5Y) neuroblastoma cells. We demonstrated that DRS can robustly identify differential expression between synthetic RNA mixes and human cell types and produce biologically relevant information. Combined with improved isoform quantification, the identification of differential expression of both genes and known and novel isoforms confirms the widespread potential for DRS in transcriptome profiling.

The key challenges with DRS are sequencing depth and correct isoform assignment. The former is due to the modest number of reads generated per DRS sequencing run (0.5–2 million on a MinION flowcell). This makes it challenging to detect and quantify many lowly expressed genes and isoforms. The challenge of correct isoform assignment has been previously noted (19,23) and is due to the high (∼10%) error rate, the large proportion of non-full-length reads and the high similarity of many gene isoforms. Although RNA extracted from 5Y cells was of very high quality (RIN > 9), it remains challenging to sequence a high proportion of full-length transcripts. DRS starts at the 3′ end of transcripts (13), therefore many incomplete reads are largely consigned to 3′UTRs and cannot specify the expressed isoform. For this reason, we do not recommend using primary alignments alone for transcriptomes with a large proportion of close secondary alignments, as this causes many reads to be specifically assigned to incorrect isoforms. Exemplifying this, >70% of reads had secondary transcriptome alignments. NanoCount helps to overcome this challenge by taking into account the unique properties of direct RNA reads. NanoCount performs a DRS specific filtering step that eliminates many incorrect alignments prior to transcript quantification, enabling it to estimate isoform abundances more reliably.

Previous studies have demonstrated DRS is a highly accurate method for quantifying expression of spike-in controls (13,23) and could identify differential gene expression in yeast (14). DGE and/or DTE have recently been reported in *C.elegans* and *Arabidopsis* with DRS (16,17). However, these have almost exclusively been identified from fold changes alone or using a relaxed statistical threshold, leaving the capability of DRS to identify differential expression in a robust manner uncertain. In addition, the human transcriptome is vastly more complex than that of simpler model organisms (50). Despite the modest read depth obtained, we found DRS was sensitive enough to detect significant expression differences in 231 genes and 333 isoforms along with 27 genes containing isoform switches during 5Y differentiation. We used our sequin controls to validate a close relationship between known and measured fold changes even if the significance threshold for DE wasn’t reached. Hence, we expect that the majority of 5Y results represent true positives, but that the results are likely missing a number of DE genes and isoforms that would be revealed with higher sequencing depths.

Despite the quantification improvements enabled by NanoCount, methodological improvements to increase the proportion of full-length transcripts will also be crucial to maximising correct transcript assignment. To this end DRS methods that enrich for RNAs with a 5′ cap have recently been introduced, allowing for the targeted sequencing of full-length RNAs with intact 3′ and 5′ ends (51–53). Another recent DRS study reported that approximately 20% of reads are truncated during sequencing due to signal noise (18). The truncation of reads due to software limitations is another obvious avenue for improvement, along with preventing RNA degradation during library preparation, for maximising full-length transcripts. The continued development of new chemistries and algorithms by ONT will also result in improved coverage towards the 5′ ends of transcripts, as well as increases in read depth and accuracy.

A key advantage of DRS is its ability to sequence transcripts as they exist in the cell, improving the detection of novel transcript isoforms and RNA modifications. In contrast, Illumina short-read sequencing can perform poorly in resolving transcript isoforms, especially those resulting from alternative splicing (8). FLAIR (32) identified ∼20 000 isoforms in 5Y cells, 34% of which were novel isoforms of known genes or potentially novel transcriptional units. By updating our transcriptome annotations to include the novel high-confidence isoforms from FLAIR, we were able to quantify them with NanoCount and detect their differential expression. Comparison to sequin controls supported the accuracy of FLAIR isoforms identified with our stringent settings, however, some caution should be taken in extrapolating the sequin results due to the lower complexity of the sequin transcriptome compared to the human one. The high number of novel transcript isoforms detected in this study and others indicates that the annotation of the human transcriptome is far from complete and that DRS combined with tools such as FLAIR and NanoCount can play an important role in discovering and quantifying these novel isoforms.

In summary, the applicability of DRS to gene expression profiling was demonstrated using synthetic controls and human cell populations. We introduced NanoCount for improved isoform quantification and show that DRS identifies biologically relevant changes in gene and isoform expression in complex transcriptomes. The long read lengths generated by the technique provide a clear advantage for isoform quantification, however several aspects of DRS require improvement before it can truly outweigh other sequencing technologies. In conclusion, DRS is a promising method to decipher the complex expression and splicing patterns that characterise the transcriptome and to identify differentially expressed isoforms contributing to development and disease.

## DATA AVAILABILITY

FAST5 and FASTQ files are publicly available to download from ENA: PRJEB39347.

All code used to perform the analysis (custom scripts, R workflows for DGE, DTE and DIU) is available on GitHub: https://github.com/josiegleeson/directRNA; https://github.com/josiegleeson/BamSlam.

NanoCount is available at: https://github.com/a-slide/NanoCount.

## Supplementary Material

gkab1129_Supplemental_Files

## Notes

Present address: Adrien Leger, Oxford Nanopore Technologies, Gosling Building, Oxford Science Park, Oxford, UK.

Present address: Tracy A. Lane, Orbit Discovery, The Schrodinger Building, Oxford Science Park, Oxford, UK.
