# Supplementary material for: Accurate expression quantification from nanopore direct RNA sequencing with NanoCount
Source: Nucleic Acids Res. 2021 Nov 25;50(4):e19. doi: 10.1093/nar/gkab1129 (PMC8886870; doi:10.1093/nar/gkab1129)
Supplement: gkab1129_Supplemental_Files [file gkab1129_supplemental_files.zip › Supplementary_Figures_Combined.pdf]

Figure S1

A

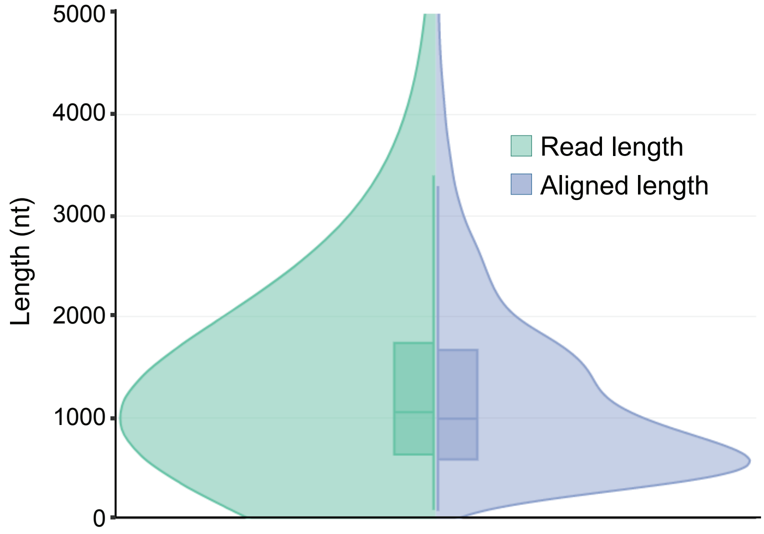

B

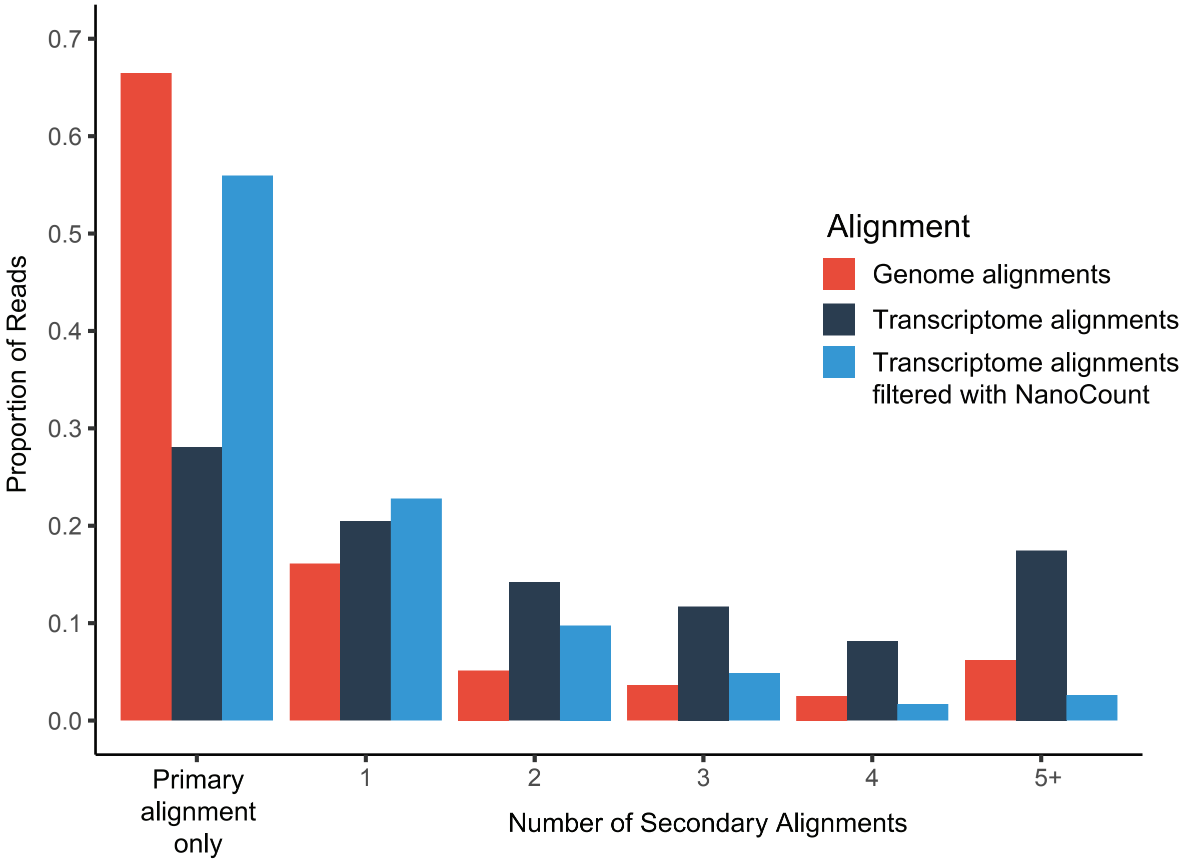

Figure S1: Alignment of nanopore dRNA reads. (A) Violin plot split into raw read lengths (green) and the length of the aligned portion of each read to the human transcriptome (purple), for SHSY5Y data. Aligned lengths are plotted for the ~98% of reads that align to the transcriptome. Box plots display length quartiles with density distributions on the outside. **(B)** Proportion of aligned reads that have one or more alignments to the genome and transcriptome. All plotted reads have a primary alignment and may also have one or more secondary alignments (see Methods). NanoCount was used to filter alignments with a 3′ greater than 50nt away from an annotated transcript end, and alignments with scores less than 95% of the highest alignment score for that read. Filtering alignments with NanoCount increases the number of reads with only a primary alignment or one secondary alignment.

Figure S2

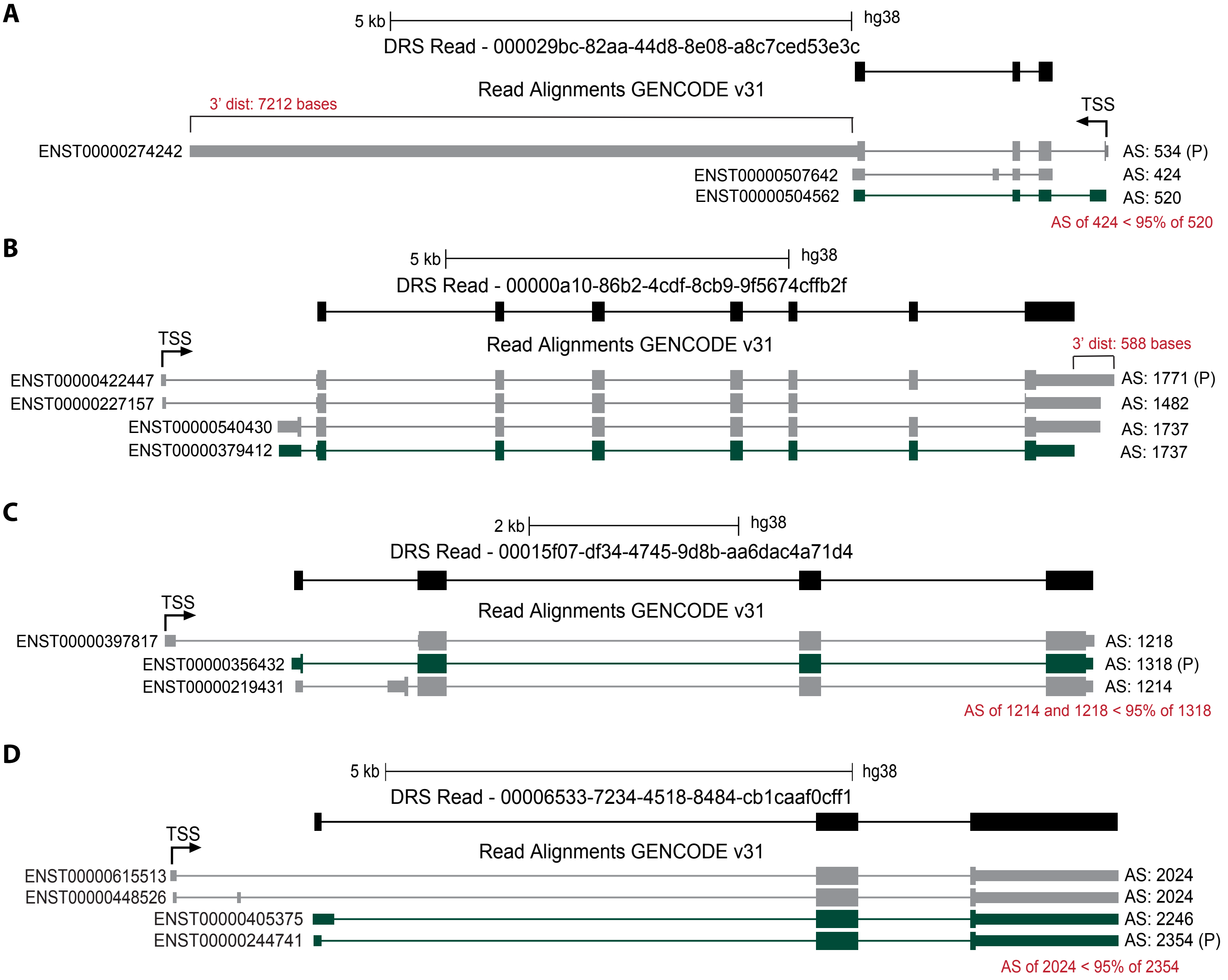

Figure S2: Read alignment filtering with NanoCount. (A-D) Examples demonstrate how NanoCount filters DRS read alignments to facilitate improved DRS isoform quantification. Reads shown in black are generated from direct RNA sequencing on 5Y cells. Minimap2 alignments reported for each read are shown in green and grey. Grey alignments do not pass the NanoCount filtering steps. Green alignments do pass the filtering steps and are subsequently used for transcript quantification by NanoCount. TSS: transcription start site. AS: alignment score. P: primary alignment. **(A)** The read's alignment to transcript ENST00000274242.10 is removed due to the 3' end distance being greater than 50 nucleotides. The read's alignment to ENST00000507642.5 is removed due to the alignment score not being within 95% of the highest alignment score. The primary alignment is not considered valid by NanoCount. **(B)** Only the alignment to transcript ENST00000379412.9 will be kept as all other possible alignments exceed the 3' end threshold. The primary alignment is not considered valid by NanoCount. **(C)** Only the primary read alignment is kept as the alignment scores for the other possible alignments are less than 95% of the highest alignment score. **(D)** Two alignments are kept, including the primary alignment. The other possible alignments are filtered out by the alignment score threshold.

Figure S3

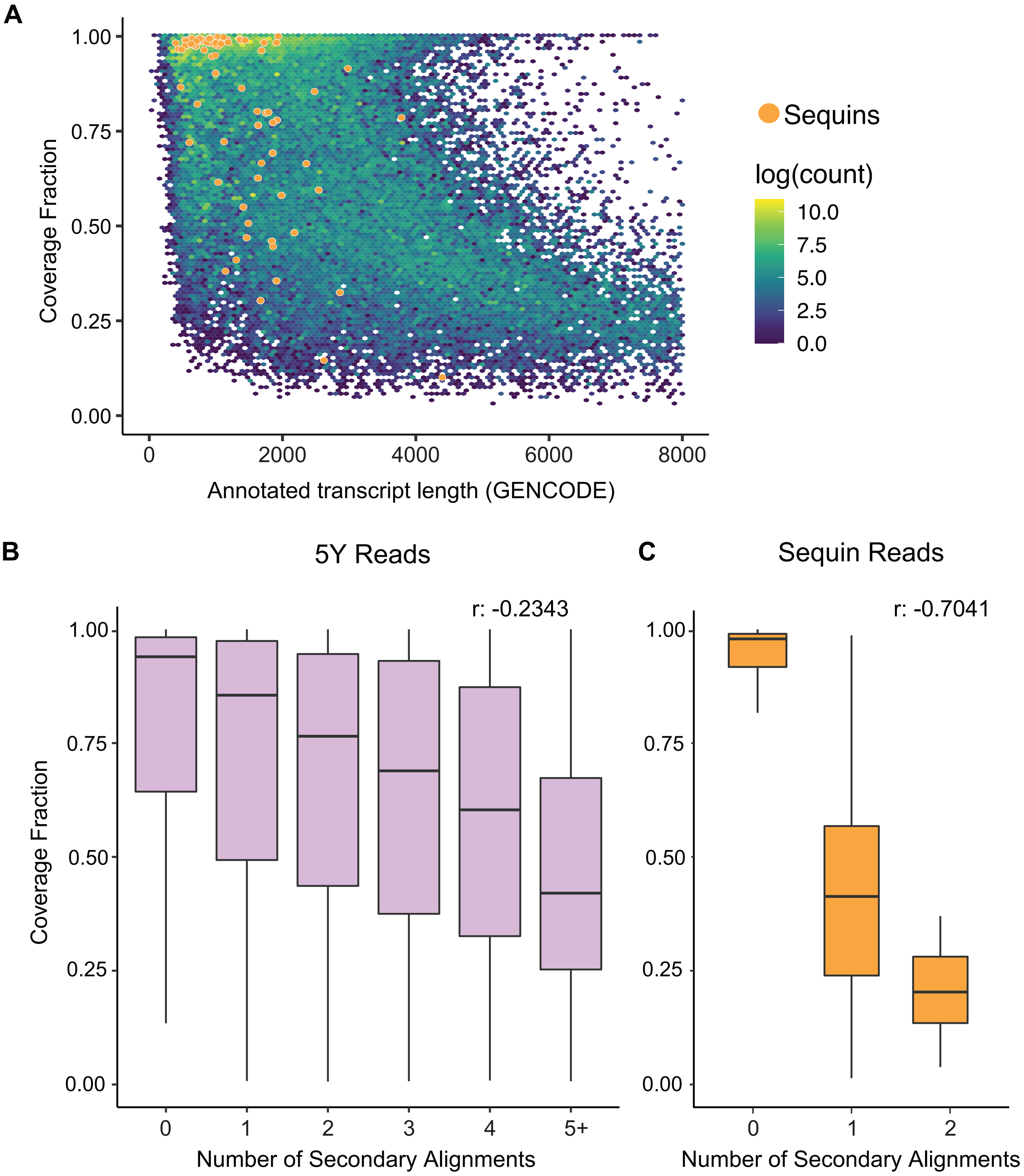

Figure S3: Transcript coverage vs transcript length. (A) Density plot showing median read coverage fraction of each Gencode transcript compared to annotated transcript length. Coverage fraction is defined as alignment length divided by known transcript length. Orange circles display median coverage fraction for each Sequin transcript. Coverage fractions shown are the median value per transcript instead of per read (as in B and Figure 1D). **(B,C)** Boxplots of the number of secondary alignments for 5Y **(B)** and sequin **(C)** reads compared with alignment coverage fraction. Spearman's correlation values (r) are shown. Zero secondary alignments indicate a read has a primary alignment only.

Figure S4

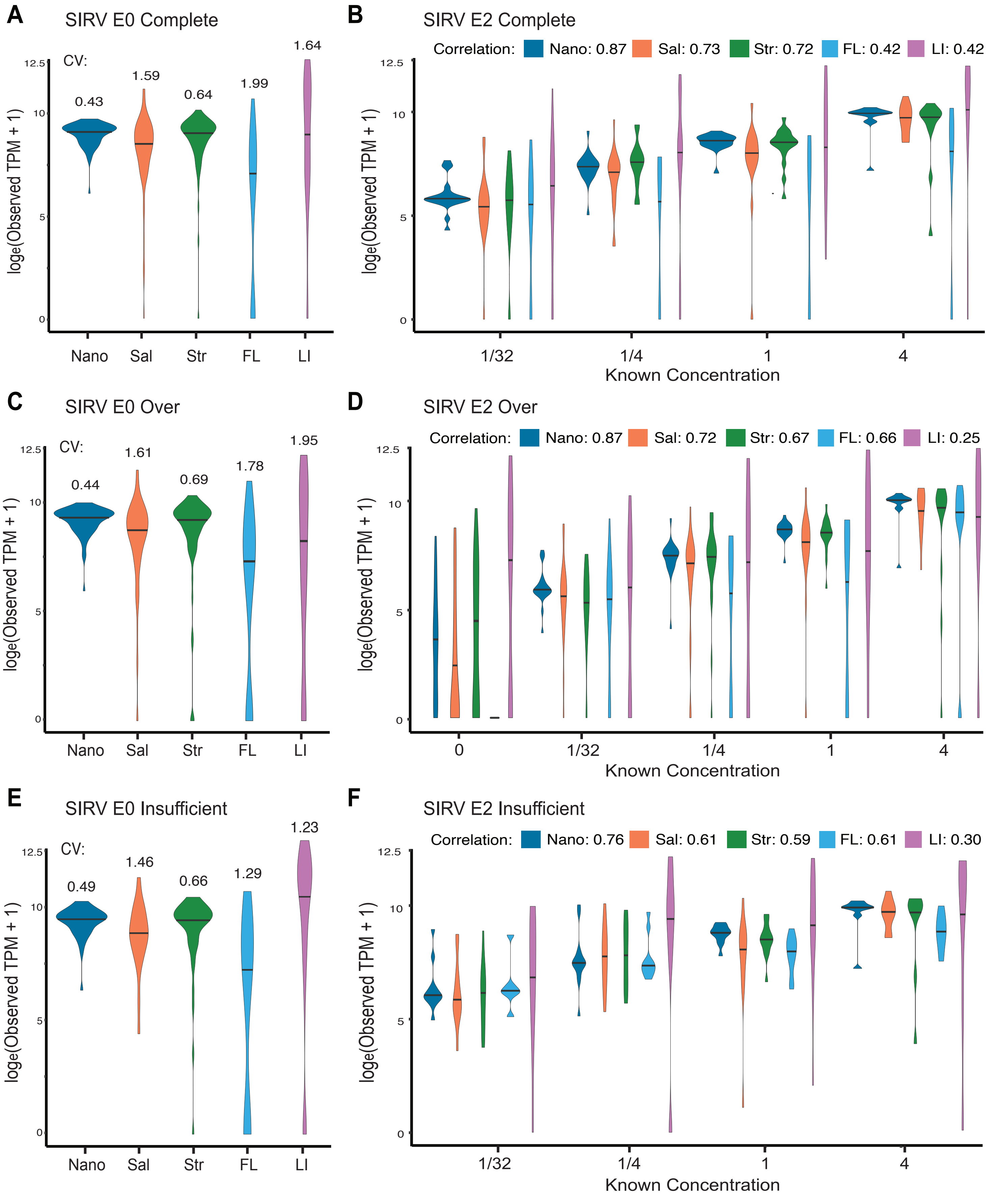

Figure S4: Quantification of SIRV spike-in control transcripts. Quantification of SIRV Mix E0 isoforms using the **(A)** Complete, **(C)** Over and **(E)** Insufficient annotations (CV = coefficient of variation). Spearman's correlations of SIRV Mix E2 isoforms with the **(B)** Complete, **(D)** Over and **(F)** Insufficient annotations. SIRV TPMs are shown from five different quantification methods: NanoCount (Nano), Salmon (Sal), StringTie (Str), FLAIR (FL) and LIQA (LI). Black bars show median values. SIRV TPMs transformed $\log_e(TPM+1)$.

Figure S5

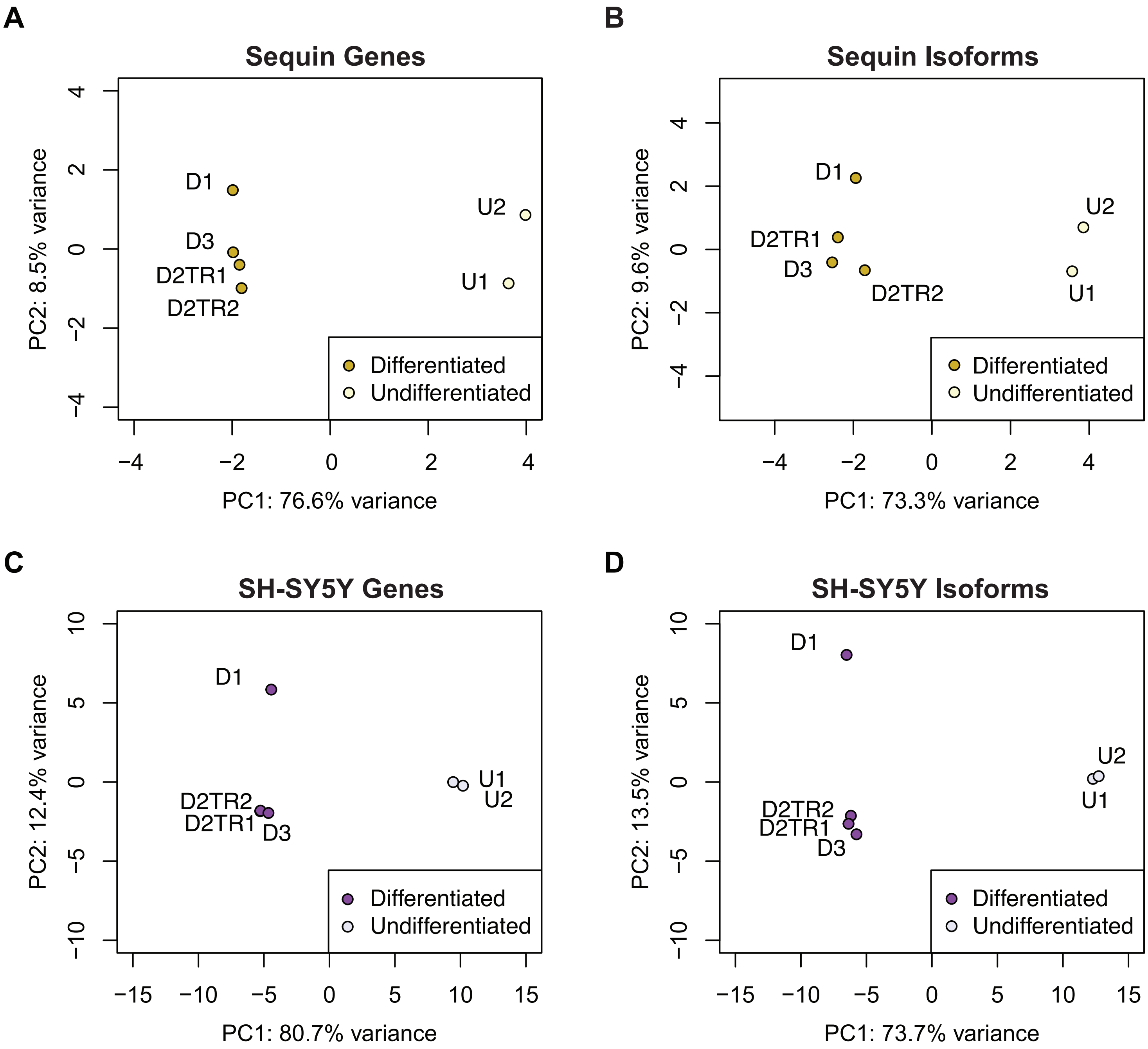

Figure S5: Principal component analysis (PCA) of Sequin **(A,B)** and SH-SY5Y **(C,D)** gene and isoform expression, including the technical replicate of differentiation sample 2 (D2). TR = Technical Replicate. All plots show the first two principal components. SH-SY5Y shows endogenous expression only. Sequins were added to undifferentiated (MixA) and differentiated (MixB) SH-SY5Y RNA and plots reflect measured abundance differences between the sequin mixes. PCAs were performed on all expressed features (no expression-based filtering of count matrices). All Sequin samples from the same mix are effectively technical replicates. SH-SY5Y technical replicates use the same polyA+ RNA with an idependant library preparation and sequencing run.

Figure S6

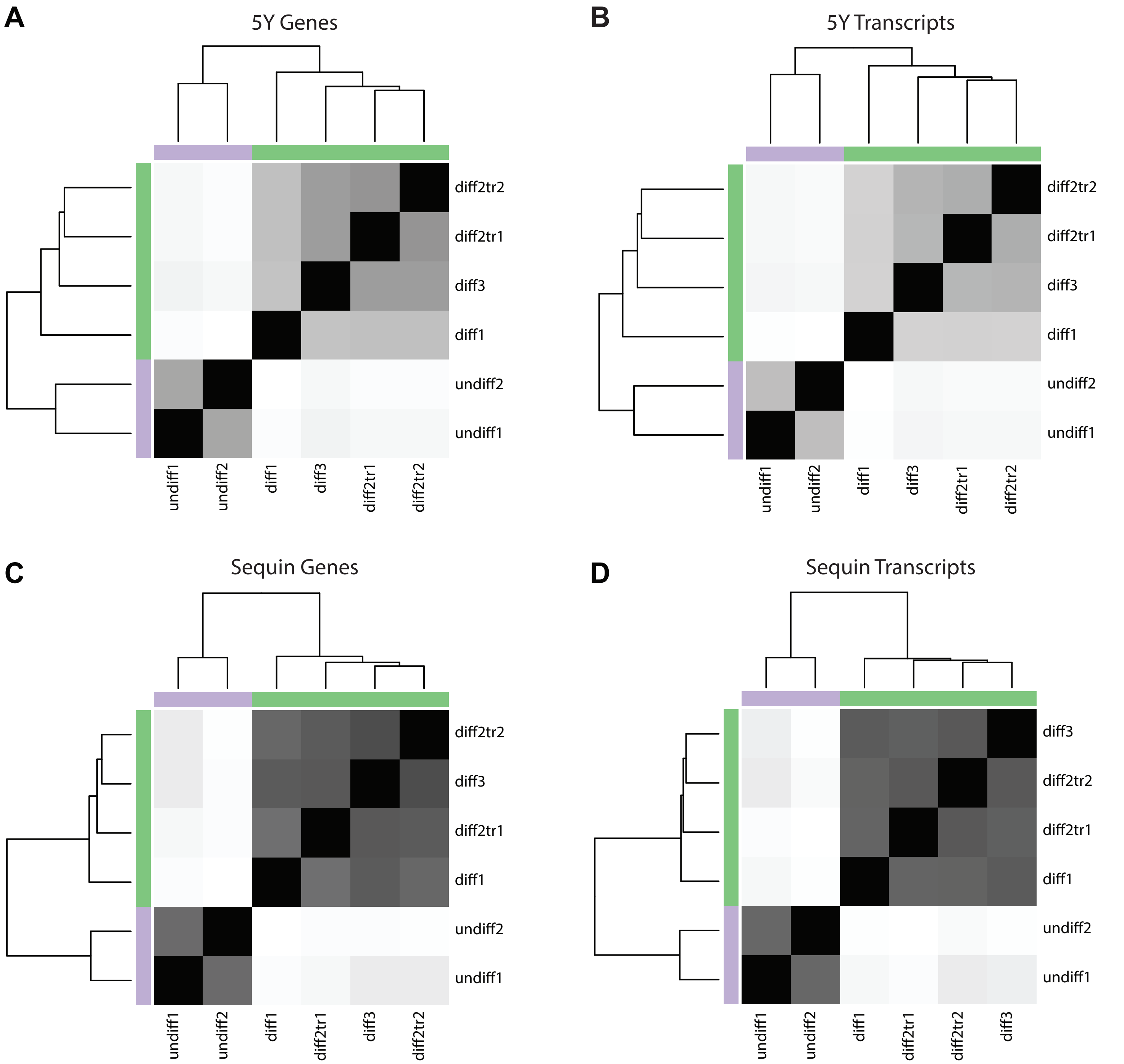

Figure S6: Heatmaps of Sequin **(A,B)** and SH-SY5Y **(C,D)** gene and isoform expression, including the technical replicate of differentiation sample 2 (D2). TR = Technical Replicate. Sequins were added to undifferentiated (MixA) and differentiated (MixB) SH-SY5Y RNA and plots reflect measured abundance differences between the sequin mixes. All Sequin samples from the same mix are effectively technical replicates. SH-SY5Y technical replicates use the same polyA+ RNA with an idependant library preparation and sequencing run, reflected in TR1 and TR2 clustering together in SH-SY5Y **(A,B)** heatmaps.

Figure S7

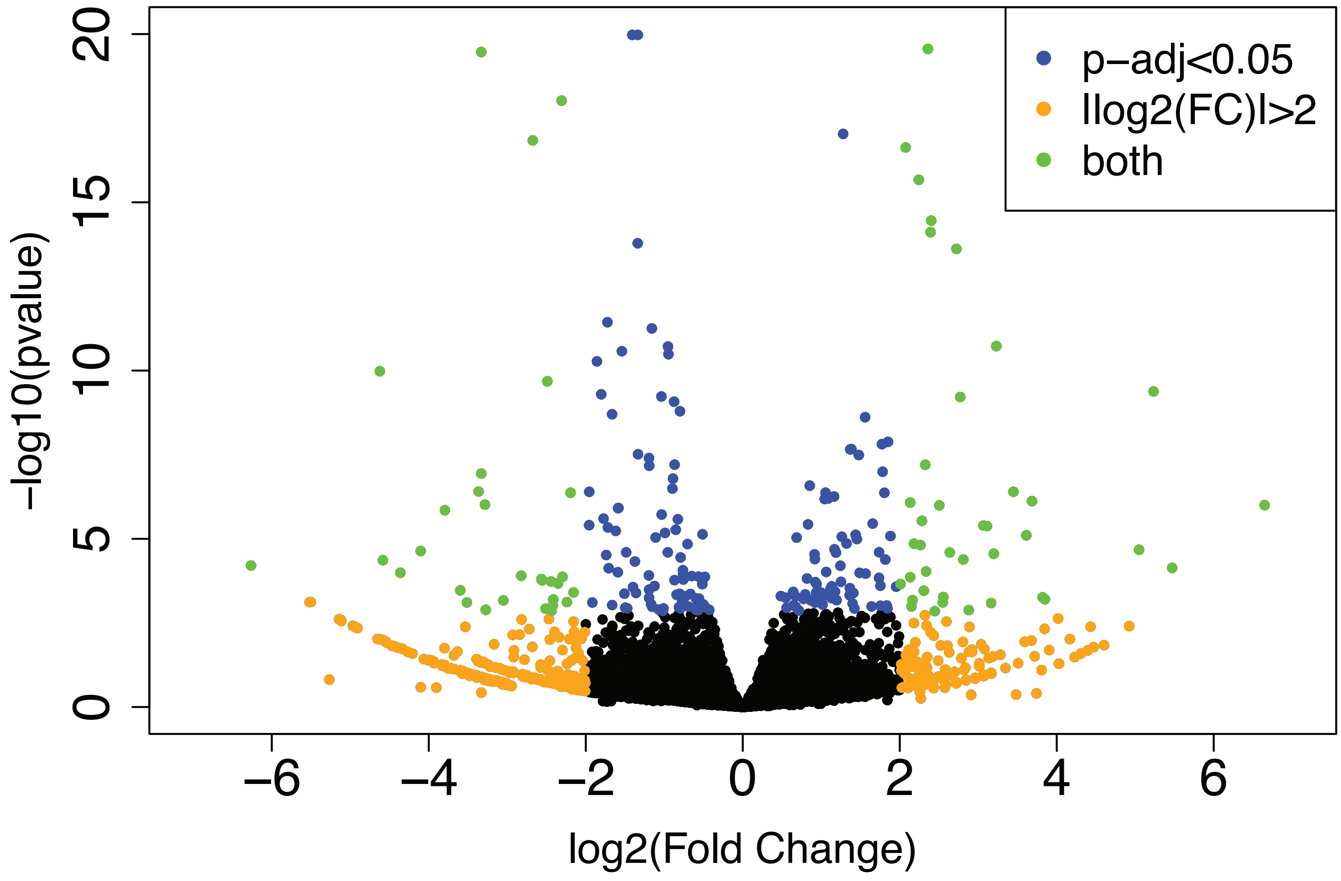

Figure S7: Differential gene expression. Volcano plot of differential gene expression between undifferentiated and differentiated SH-SY5Y cells. An adjusted p-value of <0.05 from DESeq2 (green and blue dots) was considered significant for differential expression.

Figure S8

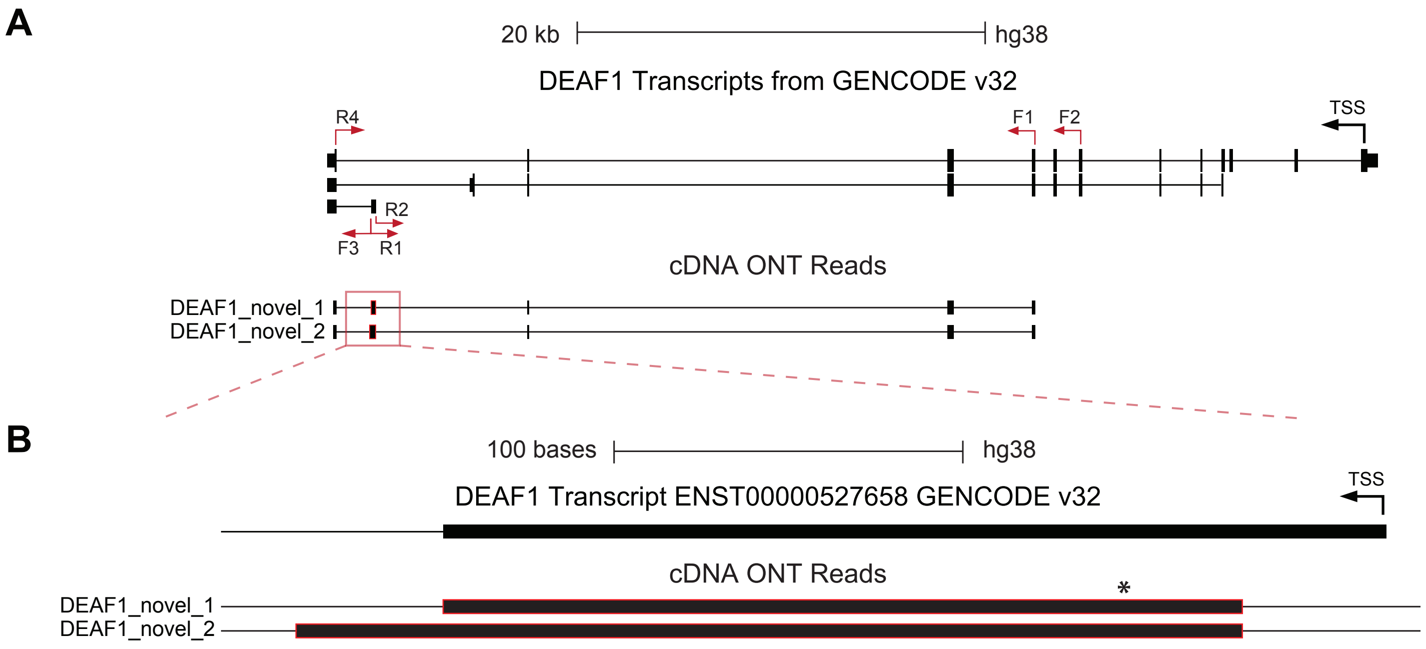

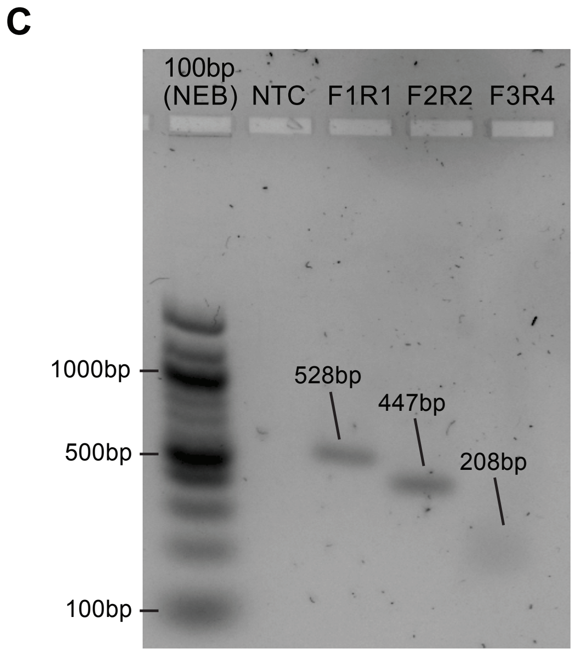

Figure S8: Validation of novel DEAF1 transcripts.
(A) UCSC Genome Browser image showing ONT cDNA sequencing of novel DEAF1 amplicons. Both amplicons show inclusion of the exon highlighted in red. Primers used to validate the novel exon are shown as red arrows.

(B) A zoomed in section of DEAF1 ONT cDNA reads, which show a novel canonical splice site at the 5′ of the exon compared to the GENCODE annotation, and two alternate canonical 3′ exon boundaries (one annotated and one novel). Both exons variants contain the same stop codon, marked with *. The position of the stop codon in the penultimate exon suggests these isoforms will be targeted for nonsense-mediated decay.

(C) Gel image of the DEAF1 PCR amplicons prepared for sequencing validations.
